# Improved Neutralisation of the SARS-CoV-2 Omicron Variant following a Booster Dose of Pfizer-BioNTech (BNT162b2) COVID-19 Vaccine

**DOI:** 10.3390/v14092023

**Published:** 2022-09-13

**Authors:** Kerri Basile, Rebecca J. Rockett, Kenneth McPhie, Michael Fennell, Jessica Johnson-Mackinnon, Jessica E. Agius, Winkie Fong, Hossinur Rahman, Danny Ko, Linda Donavan, Linda Hueston, Connie Lam, Alicia Arnott, Sharon C.-A. Chen, Susan Maddocks, Matthew V. O’Sullivan, Dominic E. Dwyer, Vitali Sintchenko, Jen Kok

**Affiliations:** 1 Centre for Infectious Diseases and Microbiology Laboratory Services, NSW Health Pathology, Institute of Clinical Pathology and Medical Research, Westmead Hospital, Westmead, Sydney, NSW 2145, Australia; 2 Centre for Infectious Diseases and Microbiology—Public Health, Westmead Hospital, Sydney, NSW 2145, Australia; 3 Sydney Institute for Infectious Diseases, The University of Sydney, Sydney, NSW 2006, Australia; 4 The Westmead Institute for Medical Research, Westmead, Sydney, NSW 2145, Australia; 5 Menzies Health Institute Queensland, Griffith University, Brisbane, QLD 4222, Australia

**Keywords:** SARS-CoV-2, COVID-19, VOC, Omicron, neutralising antibodies, immunity, vaccine, Pfizer-BioNTech BNT162b2

## Abstract

In late November 2021, the World Health Organization declared the SARS-CoV-2 lineage B.1.1.529 the fifth variant of concern, Omicron. This variant has acquired over 30 mutations in the spike protein (with 15 in the receptor-binding domain), raising concerns that Omicron could evade naturally acquired and vaccine-derived immunity. We utilized an authentic virus, multicycle neutralisation assay to demonstrate that sera collected one, three, and six months post-two doses of Pfizer-BioNTech BNT162b2 had a limited ability to neutralise SARS-CoV-2. However, four weeks after a third dose, neutralising antibody titres were boosted. Despite this increase, neutralising antibody titres were reduced fourfold for Omicron compared to lineage A.2.2 SARS-CoV-2.

## 1. Introduction

In November 2021, the SARS-CoV-2 Omicron (B.1.1.529) variant of concern (VOC) emerged in Gauteng province, South Africa, coinciding with a rapid rise in COVID-19 cases. The World Health Organization (WHO) designated Omicron a VOC on 26 November 2021, two days after its identification [1,2]. Omicron is now the predominant circulating VOC worldwide accounting for more than 98% of sequences shared on the Global Initiative on Sharing All Influenza Data (GISAID) since February 2022 [3]. Early epidemiological reports from South Africa suggest that Omicron has an increased ability to evade prior infection-induced immunity compared with the Delta and Beta VOCs [1]. Questions regarding Omicron’s ability to evade vaccine-derived immunity were also raised following transmission between two vaccinated individuals whilst in hotel quarantine [2].

Vaccination plays a key role in controlling SARS-CoV-2 transmission as well as reducing the morbidity and mortality of COVID-19. The 11 COVID-19 vaccines that have currently been granted emergency use listing (EUL) by the WHO were all developed based on ancestral SARS-CoV-2 lineages [4]. The first to receive EUL was Pfizer-BioNTech (BNT162b2), a nucleoside-modified RNA vaccine targeting the spike glycoprotein, the target of choice for many COVID-19 vaccines and therapeutics.

Despite the global rollout of novel vaccines commencing within a mere twelve months from the first reported cases of COVID-19, the viral evolution and emergence of VOCs threaten the success of vaccines in controlling the pandemic.

Each VOC contains a constellation of mutations in the spike glycoprotein with the potential for evasion of both natural and vaccine-induced immunity [5,6,7,8,9,10,11,12,13] and increased transmissibility. Omicron is characterised by over 30 non-synonymous mutations in the spike protein (e.g., E484A, K417N, P681H, N501Y, T478K), many within key epitopes that provide SARS-CoV-2 with an advantage over host immune responses [12,13,14].

Here, we present data outlining the reduced ability of sera from a COVID-19 naïve cohort collected post-two- and three-dose vaccination with BNT162b2 to neutralise Omicron compared with the Delta and wild-type lineages; however, there was improved neutralisation after the third dose of BNT162b2 or the ‘booster dose’.

## 2. Methods

### 2.1. SARS-CoV-2 Culture

Upper respiratory tract specimens collected in universal transport media where SARS-CoV-2 RNA was detected by reverse transcriptase real-time polymerase chain reaction (RT-PCR) on either a Cobas^®^ 6800 (Roche Diagnostics GmbH (Mannheim, Germany)), a BD MAX™ (Becton Dickinson (Franklin Lakes, NJ, USA)), or an in-house assay [15] were used to inoculate VeroE6-expressing transmembrane serine protease 2 (TMPRSS2) [VeroE6/TMPRSS2; JCRB1819] cells as previously outlined [16]. TMPRSS2-expressing VeroE6 cells were used for viral isolation to prevent the emergence of advantageous mutations or deletions at or near the furin cleavage site in the spike protein, which can occur when VeroE6 cells are used [17,18,19].

In brief, cells were seeded at 1–3 × 10^4^ cells/cm^2^ while in the log phase of replication with Dulbecco’s minimal essential medium (DMEM) (BE12-604F, Lonza Group AG (Basel, Switzerland)) supplemented with 9% foetal bovine serum (FBS) (10099, Gibco™, Thermo Fisher Scientific Inc. (Waltham, MA, USA)) and Geneticin™ Selective Antibiotic (G418 Sulfate) 1 mg/mL, (10131035, Gibco™, Thermo Fisher Scientific Inc. (Waltham, MA, USA)) in Costar^®^ 25 cm^2^ cell culture flasks (430639, Corning Inc.(Corning, CA, USA)) The media was changed within 12 h for inoculation media containing 1% FBS and 1% antimicrobial agents (amphotericin B deoxycholate 25 µg/mL, penicillin 10,000 U/mL, and streptomycin 10,000 µg/mL) (17-745E, Lonza Group AG (Basel, Switzerland)) to prevent microbial overgrowth and then inoculated with 500 μL of the clinical specimen into Costar^®^ 25 cm^2^ flasks. Routine testing was performed to exclude mycoplasma contamination of the cell lines and the manipulation of all SARS-CoV-2 cultures was performed under biosafety level 3 conditions.

Cultures were inspected daily for cytopathic effect (CPE); the inoculum and supernatant were sampled at 96 h for in-house quantitative reverse transcriptase real-time polymerase chain reaction (RT-qPCR) targeting the SARS-CoV-2 nucleocapsid (*N*) gene as previously described [15,20]. A decrease in the cycle threshold (Ct) from the inoculum RT-qPCR results, as well as the presence of CPE, was used to determine the propagation of SARS-CoV-2. Viral culture supernatant was harvested 96 h post-infection and clarified and then stored at −80 °C in 500 μL aliquots in 2 mL cryovials (72.694.406, Sarstedt Inc. (Nümbrecht, Germany)) until required. SARS-CoV-2 complete genomes were sequenced from the initial clinical specimen, positive culture supernatant, and post-neutralisation (72 h) to identify genomic variations that may have developed during propagation (Table S2).

### 2.2. SARS-CoV-2 Viral Load Quantitation by RT-qPCR

An RT-PCR targeting the *N* gene [15,20] was employed to estimate the viral load of the viral inoculum and post-neutralisation viral culture. Serial (10-fold) dilutions starting at 20,000 copies/µL to 2 copies/µL of a commercially available synthetic RNA control (Wuhan-1 strain, TWIST Biosciences NCBI GenBank accession MN908947.3) were used to generate standard curves and quantify the viral load of each culture extract. The mean Ct of the biological replicates was used to calculate the viral load. A positive change in the viral load between the viral inoculum (0 h) and 72 h post-neutralisation was used to indicate viral replication.

### 2.3. SARS-CoV-2 Sequencing

Tiling PCR was used to amplify the entire SARS-CoV-2 genome from the RNA extracts of the clinical specimens using primers outlined in the Midnight sequencing protocol, the viral respiratory oligo panel (RVOP, Illumina, Inc. (San Diego, CA, USA)) (Omicron), Artic v3 primers (Delta), or a previously described long amplicon methodology (wild-type) [20,21]. Each PCR included 12.5 µL Q5 High Fidelity 2 × Master Mix (New England Biolabs (Ipswich, UK)), 1.1 µL of either pool 1 or pool 2 10 µM primer master mix, and 2.5 µL of template RNA and molecular grade water was added to generate a total volume of 25 µL. Cycling conditions were initial denaturation at 95 °C for 2 min, then 35 cycles of 95 °C for 30 s, 65 °C for 2 min 45 s, and a final extension step of 75 °C for 10 min. Pool 1 and pool 2 amplicons were combined and purified with a 1:1 ratio of AMPureXP beads (Beckman Coulter (Brea, CA, USA)) and eluted in 30 µL of RNAase-free water. Purified products were quantified using Qubit™ 1 × dsDNA HS Assay Kit (Thermo Fisher Scientific Inc. (Waltham, MA, USA)) and diluted to the desired input concentration for library preparation. Sequencing libraries were prepared using Nextera XT (Illumina, Inc. (San Diego, CA, USA)) according to the manufacturer’s instructions and pooled to produce 1 × 10^6^ reads per library. Sequencing libraries were then sequenced with paired-end 76 bp chemistry on the iSeq or MiniSeq (Illumina, Inc. (San Diego, CA, USA)) platforms. 

### 2.4. Bioinformatic Analysis

Raw sequence data were processed using an in-house quality control procedure prior to further analysis as described previously [20,22]. De-multiplexed reads were quality trimmed using Trimmomatic v0.36 (sliding window of 4, minimum read quality score of 20, leading/trailing quality of 5, and minimum length of 36 after trimming) [23]. Briefly, reads were mapped to the reference SARS-CoV-2 genome (NCBI GenBank accession MN908947.3) using Burrows–Wheeler Aligner (BWA)-MEM version 0.7.17 [24], with unmapped reads discarded. The average genome coverage was estimated by determining the number of missing bases (Ns) in each sequenced genome. Variant calling and the generation of consensus sequences were conducted using iVar [25], with soft clipping over the primer regions (version 1.2.1, min. read depth >10x, quality >20, min frequency threshold of 0.1). Polymorphic sites that have previously been highlighted as problematic were monitored [26]. SARS-CoV-2 lineages were inferred using the Phylogenetic Assignment of Named Global Outbreak LINeages (Pango v.4.1.1 PLEARN-v1.12) [27,28].

### 2.5. Post Pfizer-BioNTech (BNT162b2) Vaccine Sera

Sera were collected from Australian healthcare workers caring for or handling specimens from individuals exposed to or infected with SARS-CoV-2 enrolled in the COVID Heroes Serosurvey (http://www.covidheroes.org.au; accessed on 8 December 2021). Sera were tested upon receipt with (i) an in-house immunofluorescence assay (IFA) against SARS-CoV-2-specific immunoglobulin A (IgA), immunoglobulin M (IgM), and immunoglobulin G (IgG) [29]; and (ii) an in-house indirect SARS-CoV-2 trimeric spike IgG and nucleoprotein IgG enzyme-linked immunosorbent assays (ELISA) (Table S1).

In brief, the purified recombinant antigens, trimeric spike (incorporating spike 1, receptor-binding domain (RBD), and spike 2) (REC31871-500, Native Antigen Company (Kidlington, UK)), and nucleoprotein (full-length amino acids 1-419) (REC31851-500, Native Antigen Company (Kidlington, UK)) are passively absorbed to a solid phase (Nunc-Immuno Polysorb 96-well ELISA plate (475434, Thermo Fisher Scientific Inc. (Waltham, MA, USA)). Sera samples were diluted at 1:100 with diluent buffer and added to the wells, and the plate was read in a spectrophotometer. The results were expressed semi-quantitatively as ratios, with the ratio for each sample determined by dividing the sample optical density (OD) by the calibrator OD, with the cutoffs determined by population studies. Ratios <0.9 were negative, ratios 0.9–1.1 were equivocal, and ratios > 1.1 were positive. The in-house SARS-CoV-2 trimeric spike IgG ELISA could not distinguish between spike antibodies derived from vaccinations and natural infection, and conversely, the presence of nucleoprotein antibodies indicated a naturally acquired infection.

Fourteen sera samples were included from seven participants (median age 59 years [range 34–65]; Table S1): a vaccine-naïve individual (*n* = 1), individuals who received two doses of BNT162b2 (*n* = 9), and individuals who received a third dose of BNT162b2 (booster dose) six months after the primary schedule (*n* = 4). Sera were collected one, three, and six months post-primary BNT162b2 vaccination and four weeks after the third dose of BNT162b2 (booster dose). No participants had evidence of prior SARS-CoV-2 infection, inferred by the absence of SARS-CoV-2-specific nucleocapsid antibodies on serial sampling since study enrolment. All participants completed questionnaires prior to each sera collection where they were asked to outline any testing for SARS-CoV-2 other than as part of the study and the details of any tests; no participants reported any positive results. Sera were heat-inactivated at 56 °C for 30 min to inactivate complement prior to micro-neutralisation.

### 2.6. Determination of 50% Tissue Culture Infective Dose (TCID_50_)

The viral 50% tissue culture infective dose (TCID_50_) was determined for each variant of the virus. Briefly, a passage of one aliquot of virus stock was serially diluted (1 × 10^−2^–1 × 10^−7^) in virus inoculation media. Four replicates of each virus dilution were used to inoculate VeroE6/TMPRSS2 cells at 60% confluence in Costar^®^ 24-well clear tissue culture-treated multiple-well plates (3524, Corning Inc. (Corning, NY, USA)). Plates were sealed with AeraSeal^®^ Film (BS-25, Excel Scientific Inc. (Victorville, CA, USA)) to minimise evaporation, spillage, and well-to-well cross-contamination. Plates were inspected daily for CPE and 110 μL was sampled at inoculation and 48, 72, and 96 h. Infections were terminated at 96 h based on visual inspection for CPE and used in conjunction with the RT-qPCR results to determine each isolate’s TCID_50_.

### 2.7. Micro-neutralisation Assay

VeroE6/TMPRSS2 cells were seeded with DMEM from stocks in Costar^®^ 96-well clear tissue culture-treated flat-bottom plates (3596, Corning Inc. (Corning, NY, USA)) at 40% confluence. Cells were incubated at 37°C with 5% CO_2_ for 12 h or until they reached 60% confluence. Virus stocks were diluted to 200 TCID_50_ in inoculation media. Doubling dilutions (from 1:10 to 1:320) of vaccine-naïve and post-BNT162b2 vaccination sera were added in equal proportions with the virus in a Costar^®^ 96-well plate and incubated for 60 min at 37 °C and 5% CO_2_ to enable virus neutralisation. After this incubation, the media were removed from the cell monolayer and 100 μL of fresh media was added. Each dilution of sera was performed in duplicate for each virus variant; 12 wells of uninfected cells were used per plate as a negative control. Plates were sealed with AeraSeal^®^ Film. After 60 min of viral neutralisation, a residual 110 μL was sampled from the eight naïve patient wells per virus for extraction and RT-qPCR. Plates were inspected daily for CPE with a final read at 72 h by three scientists independently. SARS-CoV-2 in-house RT-qPCR was used to quantify the viral load post-neutralisation, with 110 μL of each dilution removed at 72 h to determine viral load. An amount of 110 μL of each dilution was added to 110 μL of MagNA Pure96 External Lysis buffer (06374913001, Roche Diagnostics GmbH (Mannheim, Germany)) at a 1:1 ratio in a MagNA Pure 96-well deep-well extraction plate (06241603001, Roche Diagnostics GmbH (Mannheim, Germany)) covered with a MagNA Pure Sealing Foil (06241638001, Roche Diagnostics GmbH (Mannheim, Germany)) and was left to rest in a biosafety class-two cabinet for 10 min, a duration that has been shown to inactivate SARS-CoV-2 by an in-house verification of a published protocol [30]. The RNA was then extracted with the MagNA Pure 96 DNA and Viral NA Small Volume kit (06543588001, Roche Diagnostics GmbH (Mannheim, Germany)) on the MagNA Pure 96 Instrument (06541089001, Roche Diagnostics GmbH (Mannheim, Germany)). Median neutralisation breakpoints (CPE) were calculated using the breakpoints for the individual sera for all three SARS-CoV-2 variants.

### 2.8. Statistical Analysis

Mean neutralising antibody titres (nAbT) were evaluated and statistical significance was assessed using the *t*-test with a two-tailed hypothesis. The results were considered statistically significant at *p* <0.05. The graphs were generated using Rstudio (version 3.6.1).

## 3. Results

### 3.1. Levels of Neutralising Antibodies against Different SARS-CoV-2 Lineages

**Figure 1:**
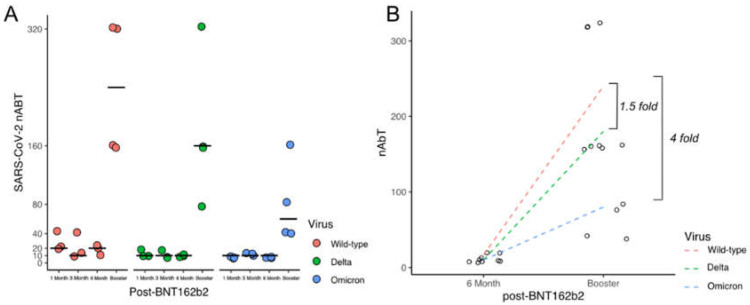
Increases in neutralising antibody titres (nAbT) four weeks after the third dose of Pfizer-BioNTech (BNT162b2). (**A**) Illustrates the neutralising antibody titre (nAbT) of SARS-CoV-2 variants Delta (green) and Omicron (blue) compared to wild-type (red, SARS-CoV-2 lineage A.2.2); black lines indicate the median titre at each time point. (**B**) Sera from four individuals collected six months after two doses of Pfizer-BioNTech (BNT162b2) and four weeks after the third dose of BNT162b2 vaccine (booster dose) demonstrate an increase in nAbT against all three variants. However, a 1.5- and 4-fold reduction in nAbT is observed for Delta and Omicron, respectively, compared to wild-type. Dashed lines depict the linear regression between individual titres (circles) pre- and post-BNT162b2 booster for each virus.

The nAbT in sera examined four weeks after the first booster dose of BNT162b2 were higher than the nAbT measured at one, three, and six months after two doses of BNT162b2 (Figure 1, Table S1). However, there was a fourfold reduction in the median nAbT against Omicron in contrast to wild-type and a 1.5-fold decrease in nAbT against the Delta VOC following the third dose. The median nAbT after one, three, and six months following two doses of the BNT162b2 vaccine for all the variants were documented with titres of <10 and <20 (Figure 1, Table S1). Trends in decreasing nAbT in sera collected one, three, and six months post-two doses of BNT162b2 were observed, which were comparable with the decreasing trends in SARS-CoV-2-specific IgG levels determined by IFA (Table S1, Figure S1). Increases in the nAbT were noted four weeks after the BNT162b2 boosters, with median titres of 240, 160, and 60 for wild-type, Delta, and Omicron, respectively. Although the median nAbT responses were lower for Delta (*p* = 0.28) and Omicron (*p* = 0.55), this reduction did not reach statistical significance.

### 3.2. Different in Vitro Infection Kinetics between SARS-CoV-2 Lineages

All three lineages demonstrated comparable TCID_50_ and viral loads at inoculation (Figure S2). Nevertheless, Omicron had a slower propagation rate with increases in the viral load not detected in the culture supernatant until 96 h post-infection (Figure S2). In contrast, the wild-type and Delta cultures showed a 4–5 log_10_ increase in the viral load 72 h post-inoculation (Figure S3).

## 4. Discussion

Omicron has rapidly evolved to encode over 30 protein substitutions in the SARS-CoV-2 spike protein, raising grave concerns for the effectiveness of COVID-19 vaccines, which were designed using ancestral viruses. Although reductions in the nAbT are noted when human sera from double-dose BNT162b2-vaccinated individuals were challenged with Omicron, sera after a third dose (booster dose) neutralised SARS-CoV-2. The observation of delays in Omicron growth in vitro suggests that these mutational changes could negatively affect viral fitness. Further investigations are required to determine if the cell infection dynamics are due to the reduced efficiency in the viral entry or host cell egress, although others have noted that ACE2 is still required for efficient Omicron propagation in cell cultures [31]. Given the longer growth times, understanding the viral kinetics of Omicron infection is important to guide infection control practices and individual patient management, as the duration of infectivity, peak viral load, and shedding may be different. Our findings also support the notion that booster doses of vaccines are likely to be required to minimise SARS-CoV-2 transmission given that BNT162b2 is highly effective in preventing severe SARS-CoV-2 infection and mortality, particularly in those with waning immunity post-primary vaccination [32]. Despite the limited sample number, this study’s strength is the serial sera samples from four participants collected pre-and post-booster with BNT162b2. Our study also lacked samples from persons previously infected with SARS-CoV-2, but previous studies have demonstrated comparable antibody responses to vaccine recipients after six months [29]. The absence of SARS-CoV-2 infection in any of the participants contributing to the sera in the present study also allowed a more accurate interpretation of the serology results that were unaffected by previous infections. Our study did not assess the durability of the antibody responses following the booster BNT162b2 vaccination, and the results herein may not be generalizable to other VOCs. In addition, there may also be VOC-specific vaccines that confer greater and longer-lasting protection against SARS-CoV-2 infection in the future. Laboratory variables such as the cell lines, SARS-CoV-2 variants, age groups, timing of the sera collection (in relation to infection and/or vaccination), and test methods used (TCID_50_; incubation times; type of neutralisation test, e.g., focus, plaque, or micro-neutralisation) need to be considered when interpreting nAbT studies.

## 5. Conclusions

These initial findings suggest that Omicron can be neutralised by sera collected from BNT162b2 recipients, optimally after three vaccine doses. However, the ability to neutralise Omicron is reduced, suggesting that there may be high incidences of breakthrough infections or reinfections despite vaccine boosters. Further studies with larger and targeted cohorts (e.g., the young, elderly, or immunocompromised) are warranted, alongside investigations into Omicron’s evasiveness to T-cell responses and primary and boosted vaccine effectiveness.

## Data Availability

Not applicable.

## Supplementary Materials

The following supporting information can be downloaded at https://www.mdpi.com/article/10.3390/v14092023/s1, Table S1: Study Participant Demographic and In-house SARS-CoV-2 Immunofluorescence (IFA) and Neutralising Antibody Titres (nAbT); Figure S1: Waning levels of SARS-CoV-2-specific IgG at 1, 3, and 6 months following two doses of the third dose of Pfizer-BioNTech (BNT162b2), which are boosted 4 weeks after the 3 BNT162b2 doses; Figure S2: Infection kinetics of SARS-CoV-2 lineages propagated in VeroE6/TMPRSS2 cells; Figure S3: Change in SARS-CoV-2 viral load 72 h post-neutralisation with sera collected post-Pfizer-BioNTech (BNT162b2) challenged with SARS-CoV-2 VOCs Delta and Omicron compared to wild-type (lineage A.2.2) virus; Table S2: Non-Silent mutations in SARS-CoV-2 isolates used in micro-neutralisation experiments.
