# Supplementary material for: Improved Neutralisation of the SARS-CoV-2 Omicron Variant following a Booster Dose of Pfizer-BioNTech (BNT162b2) COVID-19 Vaccine
Source: Viruses. 2022 Sep 13;14(9):2023. doi: 10.3390/v14092023 (PMC9501619; doi:10.3390/v14092023)
Supplement: Supplementary file 1 [file viruses-14-02023-s001.zip › viruses-1880987-supplementary.pdf]

Supplementary Material

Improved Neutralisation of the SARS-CoV-2 Omicron Variant Following a Booster Dose of Pfizer-BioNTech (BNT162b2) COVID-19 Vaccine

Kerri Basile [1,*], Rebecca J Rockett [2,3], Kenneth McPhie [1,4], Michael Fennell [1], Jessica Johnson-Mackinnon [2], Jessica E Agius [2], Winkie Fong [2], Hossinur Rahman [1], Danny Ko [1], Linda Donavan [1], Linda Hueston [1,5], Connie Lam [2], Alicia Arnott [1], Sharon C-A Chen [1,2,3], Susan Maddocks [1], Matthew V O'Sullivan [1,2,3], Dominic E Dwyer [1,2,3], Vitali Sintchenko [1,2,3] and Jen Kok [1,2,*]

Table S1. Study Participant Demographic and In-house SARS-CoV-2 Immunofluorescence (IFA) and Neutralising Antibody Titres (nAbT)

Sera	Sex	Age (yrs)	Timing post BNT162b2 (months)	SARS-CoV-2 ELISA		SARS-CoV-2 IFA			Virus Lineage nAbT		
				N ratio	S ratio	IgG	IgA	IgM	Wildtype	Delta	Omicron
**5**	M	65	1	0.13	12.3	320	<10	<10	40	20	<10
**3**	M	49	1	0.5	11.87	320	<10	<10	20	<10	<10
**3**	M	49	3	0.29	8.96	40	<10	<10	<10	<10	<10
**9**	F	59	1	0.17	11.92	160	<10	<10	<10	<10	<10
**9**	F	59	3	0.16	11.62	80	<10	<10	<10	<10	<10
**9**	F	59	Booster (1M)	0.14	12.3	1280	<10	<10	320	320	160
**1**	F	36	Naive	0.58	0.64	<10	<10	<10	<10	<10	<10
**1**	F	36	3	0.16	12.3	80	<10	<10	40	20	<10
**14**	F	34	6	NA	NA	80	<10	<10	20	<10	<10
**14**	F	34	Booster (1M)	NA	NA	640	<10	<10	160	160	40
**15**	M	63	6	0.1	6	40	<10	<10	<10	<10	<10
**15**	M	63	Booster (1M)	0.17	13	640	20	<10	160	80	80
**16**	M	59	6	0.4	7.9	40	<10	<10	20	<10	<10
**16**	M	59	Booster (1M)	1.01	10.51	160	20	<10	320	160	40

Key: BNT162b2 – Pfizer-BioNTech (BNT162b2) vaccination; Booster (1M) – 4 weeks after 3rd booster dose of BNT162b2; Delta – Delta (B.1.617.2) lineage; ELISA – Enzyme linked immunosorbent assay; F – female; IFA – Immunofluorescence Assay; IgA – Immunoglobulin A; IgG – Immunoglobulin G; IgM – Immunoglobulin M; M – male; NA – Not available; N – nucleoprotein; nAbT – Neutralising antibody titre; Omicron – Omicron (B.1.1.529) lineage; S – Trimeric Spike; Wild-type – Wildtype (A.2.2) lineage.

Figure S1. Waning levels of SARS-CoV-2-specific IgG 1, 3 and 6 months following two doses of Pfizer-BioNTech (BNT162b2) which are boosted 4 weeks after the 3rd BNT162b2 dose (booster dose)

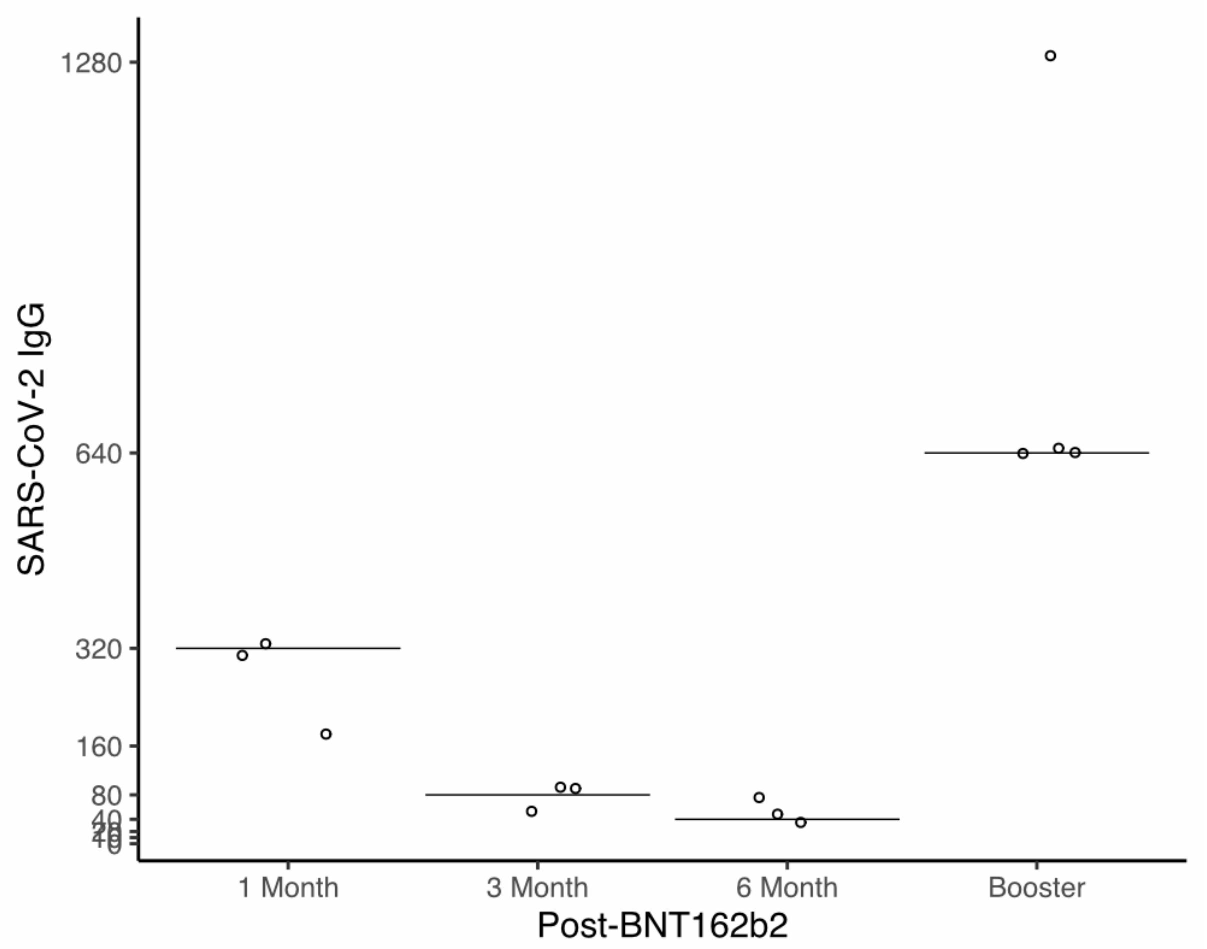

Figure S1. Illustrates levels of SARS-CoV-2-specific IgG 1, 3 and 6 months following two doses of Pfizer-BioNTech (BNT162b2) and 4 weeks after the 3rd dose (boosting dose) of BNT162b2. Levels have been determined using an immunofluorescence assay (IFA), black lines depict the median IgG level for each timepoint.

Figure S2. Infection kinetics of SARS-CoV-2 lineages propagated in VeroE6/TMPRSS2 cells

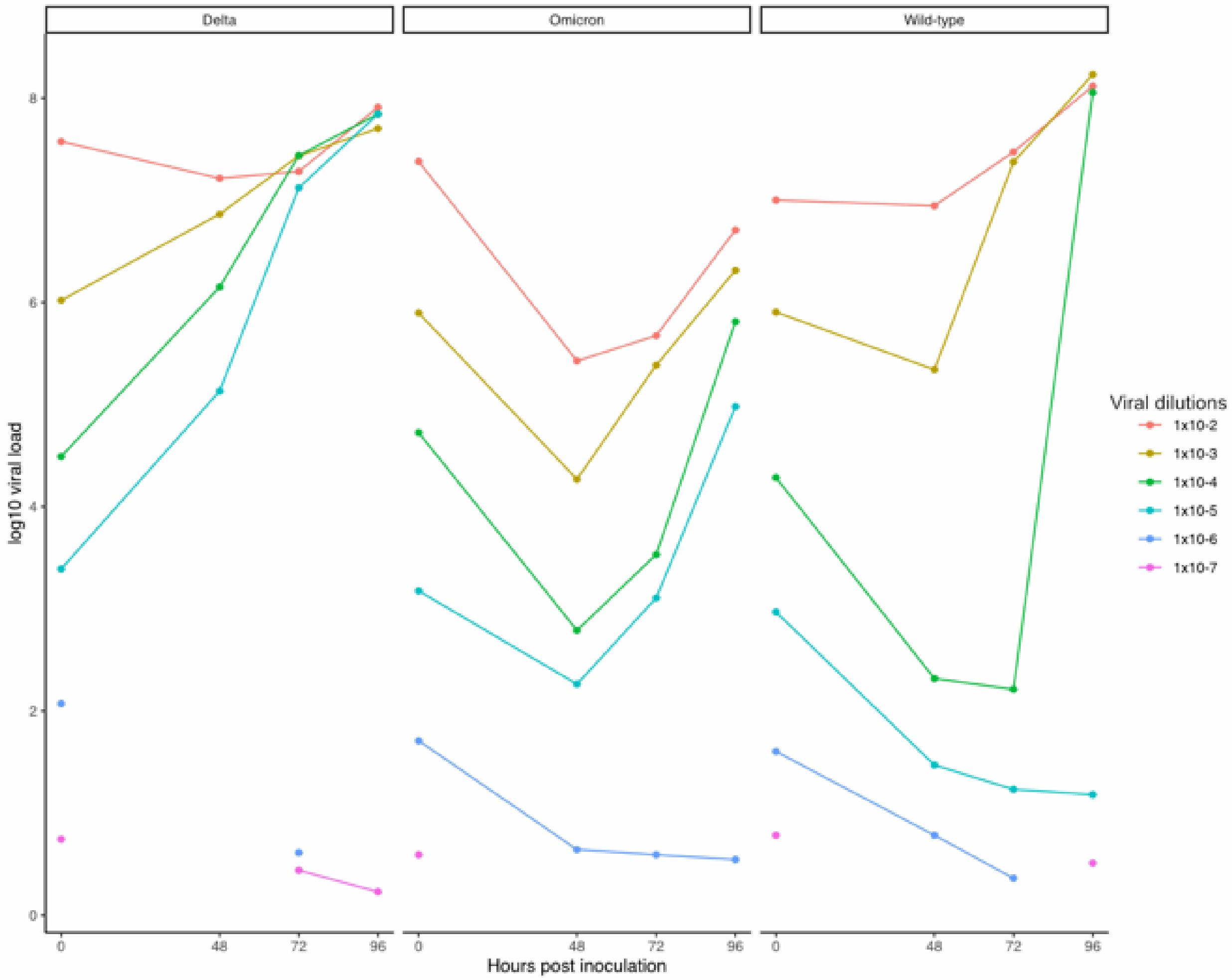

Figure S2. Illustrates infection kinetics of SARS-CoV-2 variants propagated in VeroE6/TMPRSS2 cells. Circles represent the viral load of serial dilutions of virus quantified at inoculation, 48, 72 and 96 hrs post-infection determined by SARS-CoV-2 in-house quantitative reverse transcriptase real time polymerase chain reaction (qRT-qPCR) targeting the *N*-gene

Figure S3. Change in SARS-CoV-2 viral load 72 hours post-neutralisation with sera collected post- Pfizer-BioNTech (BNT162b2) challenged with SARS-CoV-2 VOCs Delta and Omicron compared to wildtype (lineage A.2.2) virus

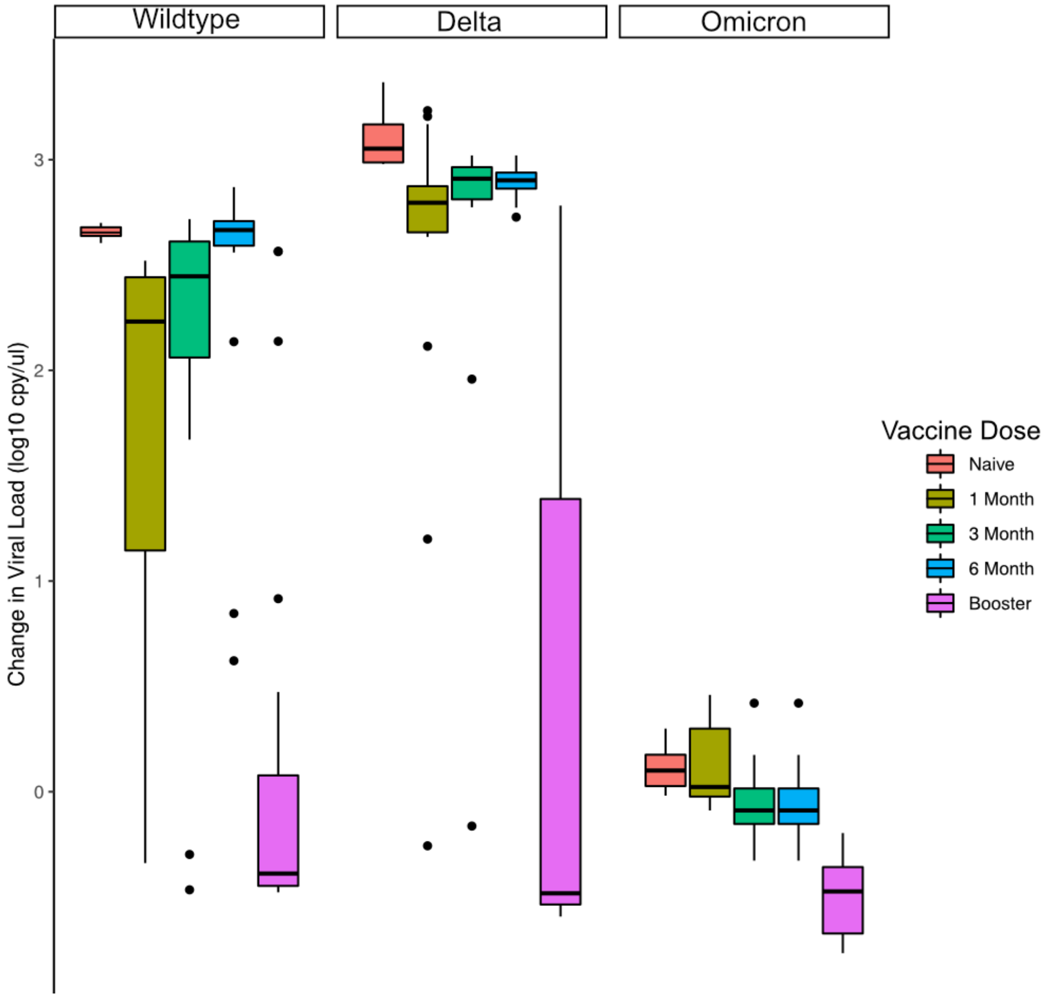

Figure S3. Illustrates replication of SARS-CoV-2 variants post-neutralisation when neutralised sera collected at different intervals post- Pfizer-BioNTech (BNT162b2). Results are reported in box−whiskers plots as medians and upper and lower quartiles.

Table S2: Non-Silent mutations in SARS-CoV-2 isolates used in Micro-neutralisation experiments.

**SARS-CoV-2 Virus**	**Non-silent Mutations**	**Pango Lineage (GISAID accession)**
**Omicron**	NSP3:K38R, NSP3:S1265(del), NSP3:A1892T, NSP4:T492I, NSP5:P132H, NSP6:L105(del), NSP6:I189V, NSP12b:P314L, NSP14:I42V, S:A67 (del), S:I68 (del), S:T95I, S:G142 (del), S:I210 (ins), S:N211(del), S:G339D, S:S371L, S:S373P, S:S375F, S:K417N, S:N440K, S:G446S, S:S477N, S:T478K, S:E484A, S:Q493R, S:G496S, S:Q498R, S:N501Y, S:Y505H, S:T547K, S:D614G, S:H655Y,S:N679K, S:P681H, S:N764K, S:D796Y*, S:N856K*, S:Q954H, S:N969K, S:L981F, E:T9I, M:D3G, M:Q19E, M:A63T, N:P13L*, N:E31(del)*, N:A134V*, N:RG203KR, ORF10:R24C	BA.1.17 (EPI_ISL_7987968)
**Delta**	NSP3:H323Y, NSP3:A488S, NSP3:P1228L, NSP3:P1469S, NSP4:V167L, NSP4:T492I, NSP6:T77A, NSP12b:P314L, NSP12b:G662S, NSP13:P77L, NSP14:A394V, NSP16:Q238H, S:T19R, S:T95I*, S:G142D*, S:E156(del), S:L452R, S:T478K, S:D614G, S:P681R, S:D950N,ORF3a:S26L,M:I82T, ORF7a:G70(del), ORF7a:V82A, ORF7a:T120I, ORF7b:T40I, ORF8:D119, N:D63G, N:R203M, N:G215C, N:D377Y	AY.39.1 (EPI_ISL_3398616)
**Wildtype**	NSP4:F308Y, ORF3a:G196V, ORF8:L84S, N:P13L, N:S197L	A.2.2 (EPI_ISL_427714)

* Missing sequence data over mutations due to mismatches in primer sequences in one of the three SARS-CoV-2 lineages sequenced (Clinical specimen, 96 hrs post viral culture. 72 hrs post-neutralisation).
